# Predictors of functional outcome vary by the hemisphere of involvement in major ischemic stroke treated with intra-arterial therapy: a retrospective cohort study

**DOI:** 10.1186/1471-2377-10-25

**Published:** 2010-04-23

**Authors:** Albert J Yoo, Javier Romero, Reza Hakimelahi, Raul G Nogueira, James D Rabinov, Johnny C Pryor, R Gilberto González, Joshua A Hirsch, Pamela W Schaefer

**Affiliations:** 1 Neuroradiology Division, Department of Radiology, Massachusetts General Hospital, Boston, Harvard Medical School, Boston, MA 02114, USA; 2 Interventional Neuroradiology Division, Department of Radiology, Massachusetts General Hospital, Boston, Harvard Medical School, Boston, MA 02114, USA; 3 Department of Neurology, Massachusetts General Hospital, Boston, Harvard Medical School, Boston, MA 02114, USA

## Abstract

**Background:**

Conflicting data exists regarding the effect of hemispheric lateralization on acute ischemic stroke outcome. Some of this variability may be related to heterogeneous study populations, particularly with respect to the level of arterial occlusion. Furthermore, little is known about the relationship between stroke lateralization and predictors of outcome. The purpose of this study was to characterize the impact of stroke lateralization on both functional outcome and its predictors in a well-defined population of anterior circulation proximal artery occlusions treated with IAT.

**Methods:**

Thirty-five consecutive left- and 35 consecutive right-sided stroke patients with intracranial ICA and/or MCA occlusions who underwent IAT were retrospectively analyzed. Ischemic change on pre-treatment imaging was quantified. Reperfusion success was graded using the Mori scale. Good outcome at three months was defined as an mRS ≤ 2. Left- and right-sided strokes were compared for outcome and its predictors.

**Result:**

Of 70 patients with median NIHSS score of 18 (IQR, 14-21), 19 (27.1%) had a good outcome. There were 21 terminal ICA and 49 MCA occlusions. There was no difference in the rate of good outcomes between left- (n = 9) and right-sided (n = 10) strokes (p = 0.99). There were no significant differences in occlusion level, age, ischemic change on initial imaging and degree of reperfusion between left- and right-sided strokes. Left-sided strokes had higher baseline NIHSS scores (p = 0.02) and lower admission SBP (p = 0.009). Independent predictors of outcome for left-sided strokes were NIHSS (p = 0.0002) and reperfusion (p = 0.006), and for right-sided strokes were age (p = 0.002) and reperfusion (p = 0.003). In univariate analysis, pre-treatment ischemic change on NCCT was associated with outcome only for left-sided strokes (p = 0.05).

**Conclusions:**

In anterior circulation proximal artery occlusions treated with IAT, hemispheric lateralization influences the clinical and imaging predictors of outcome. Most notably, NIHSS predicts outcome only for the left-sided strokes in this population. This finding has important implications for outcome prediction in the acute setting and indicates a need for stroke severity scales more sensitive to right hemispheric deficits.

## Background

Numerous studies have explored the differences between left- and right-hemispheric strokes, particularly with regards to functional outcome [1-3], NIHSS score [4,5], and imaging findings [4-6]. While certain findings are widely accepted, such as weighting of the NIHSS toward left-hemispheric function, conflicting data exists on the impact of hemispheric lateralization on clinical outcome[3]. Some variability may be related to differences in study populations. In particular, many studies do not adjust for the level of arterial occlusion, which is noted to be a major determinant of outcome[7].

Intracranial PAO represents a relatively homogeneous population of AIS that accounts for the majority of stroke-related morbidity and mortality[8]. IAT is more effective than intravenous thrombolysis for these proximal occlusions [9-11], and this recognition has led to a dramatic growth in the utilization of endovascular therapies[12]. Several studies, including one randomized, controlled study, have explored the factors predictive of outcome following IAT[9,11,13-20]. None have analyzed these variables stratified by the side of involvement.

Baseline NIHSS score is widely recognized to be a strong predictor of clinical outcome in acute ischemic stroke[13,15,21,22]. Multiple studies comparing left- and right-sided strokes have demonstrated that the NIHSS underestimates stroke severity in right-sided strokes [4-6], and this difference is most pronounced in large strokes secondary to PAO[4]. Yet, no studies have explored whether the prognostic ability of the NIHSS is influenced by the side of stroke involvement in this population.

Our purpose was to characterize the effect of hemispheric lateralization on functional outcome and the clinical and imaging predictors of outcome in acute ischemic stroke patients with PAO undergoing IAT.

## Methods

### Patient selection

This retrospective cohort study examined 35 consecutive left-hemispheric and 35 consecutive right-hemispheric stroke patients with anterior circulation PAO who underwent IAT at Massachusetts General Hospital from June 2004 to August 2008. Inclusion criteria were: (1) acute occlusion of the intracranial ICA and/or proximal MCA, including stem (M1) and proximal branch lesions (M2), on CTA; (2) treatment with IAT; and (3) available clinical follow-up at three months. Medical records were reviewed for clinical data. Our institutional review board approved the study.

### Imaging acquisition

All subjects underwent NCCT followed by CTA of the head and neck performed on a 16- or 64-slice multi-detector helical scanner (GE Medical Systems, Waukesha, WI). NCCT was performed with 120-140kVp, 170mA, and 5-mm axial slice thickness. For CTA, nonionic low-osmolar iodinated contrast media (Isovue, 370 mg iodine/mL; Bracco Diagnostics, Princeton, NJ) was injected via a peripheral intravenous catheter at 3.5 cc/s for 100 cc, followed by 40 cc saline at 4.0 cc/s. Images were acquired from the vertex to the aortic arch after an 8-10s delay, using 140 kVp, auto-mA (300-715 mA), 0.5s rotation time, 1.25 mm helical thickness, 0.625 mm interval, 0.938:1 pitch, 9.37 mm/rotation speed, 22 cm FOV. CTA images were reconstructed in standard algorithm at 2.5 mm.

### Treatment

#### Intra-arterial reperfusion therapy

Inclusion criteria are:(1) proximal artery occlusion (ICA, MCA M1 or M2 branches) on CTA; (2) NCCT without hemorrhage; (3) NCCT with hypodensity less than one-third of the MCA territory, or a PWI-DWI mismatch >20% with DWI abnormality <1/3rd of the MCA territory; and (4) ability to navigate a microcatheter to the thrombus within eight hours from symptom onset. Exclusion criteria are comparable to IV tPA criteria[23].

Mechanical/pharmacological treatments included the MERCI Retriever (Concentric Medical Inc., Mountain View, CA), microwire disruption, balloon angioplasty, stent placement and IA urokinase infusion (5000U/cc, maximum dose 750,000U). Some patients also received IV tPA (bridging therapy): 0.9 mg/kg of IV tPA to a maximum of 90 mg[24].

Reperfusion was graded on post-treatment digital subtraction angiography with the Mori scale: grade 0--complete occlusion; 1--distal movement of thrombus without reperfusion; 2--reperfusion in <50% of the ischemic area; 3-->50% reperfusion; 4--complete recanalization/reperfusion[21]. We defined reperfusion as a Mori score ≥ 2. Time from symptom onset to reperfusion was recorded. Time to procedure termination was recorded for nonrecanalizers (Mori 0-1).

### Imaging analysis

The ASPECTS grading system was applied to the pre-treatment NCCT and CTA-SI for each patient[25]. The ten brain regions (caudate, lentiform nucleus, internal capsule, insula, and six cortical regions) used to calculate the ASPECTS score were inspected using the entire set of images for each study. One point was deducted for each affected region from a total possible score of ten. Therefore, lower scores indicated more extensive ischemic change. Readers were blinded to all clinical information except for stroke side. Window width and level settings were selected to optimize contrast between normal and hypodense (ischemic) brain parenchyma. All imaging was evaluated independently by three neuroradiologists (AY, JR, PS), and differences were resolved by consensus.

Follow-up imaging from 12-72 hours after IAT was reviewed to evaluate for hemorrhagic transformation based on ECASS definitions: HI1 (small petechiae without mass effect), HI2 (more confluent petechiae without mass effect), PH1 (<30% of infarct with mild mass effect), PH2 (>30% of infarct with significant mass effect)[26].

### Clinical scoring

The baseline NIHSS score was used to assess the neurologic status of stroke patients in the emergency department. Three-month mRS, the primary clinical endpoint, was assessed by one of the stroke neurologists or neurointerventionalists, who were not blinded to clinical information. An mRS ≤ 2 was considered a good outcome.

### Statistical analysis

Univariate analysis of clinical, imaging and outcome data was performed comparing left- and right-hemispheric stroke patients. Subsequently, univariate analysis comparing good and poor outcome was performed for each hemisphere. The Fisher's exact test was used for categorical variables. The Student's t test was used for normally-distributed continuous variables. The Mann-Whitney U test was used for ordinal data. A stepwise multiple logistic regression was performed to identify independent predictors of outcome. Normality was tested with the Kolmogorov-Smirnov test. Statistical analysis was performed utilizing the SAS 9.1 software package (SAS Institute Inc., Cary, NC) and MedCalc^® ^10.0 Software.

## Results

### Baseline characteristics and clinical outcome: entire cohort

Table 1 lists the baseline and post-treatment characteristics of the entire study population. Thirty-eight of the 70 patients were females (54.3%); mean age was 67.1 ± 18.4 years; median NIHSS score was 18 (interquartile range IQR, 14-21). There were 21 terminal ICA occlusions, all extending into the proximal MCA. There were 49 MCA occlusions: 40 involved the M1 segment and 9 involved an M2 segment. The median NCCT ASPECTS was 8 (IQR, 6-9). The median CTA-SI ASPECTS was 5 (IQR, 3-7).

**Table 1:** Baseline and Post-Treatment Clinical and Imaging Characteristics, Entire Cohort (n = 70 patients)

Age, y, mean ± SD (range)	67.1 ± 18.4 (18-93)
Female, no. (%)	38 (54.3)
Baseline NIHSS score, median (IQR), range	18 (14-21), 4-30
Admission Glucose level, mean ± SD	142.5 ± 44.0
Admission SBP, mean ± SD	158.4 ± 32.2
Atrial fibrillation, no. (%)	32 (45.7)
Diabetes, no. (%)	18 (25.7)
IV tPA treatment, no. (%)	29 (41.4)
Time to imaging^a^, minutes, mean ± SD	195.6 ± 137.6
Baseline NCCT ASPECTS, median (IQR), range	8 (6-9), 2-10
Baseline CTA-SI ASPECTS, median (IQR), range	5 (3-7), 0-10
ICA:M1:M2, no.	21:40:9
Time to vessel opening^a^, minutes, mean ± SD	411.2 ± 153.2
Mori reperfusion score, median (IQR), range	2 (1-3)
Post-treatment hemorrhage, no. (%)	39 (58.2)^b^
Good 3-month outcome (mRS ≤ 2), no. (%)	19 (27.1)

Abbreviations: NIHSS, National Institutes of Health Stroke Scale; SBP, systolic blood pressure; IV tPA, intravenous tissue plasminogen activator; NCCT, noncontrast head CT; CTA-SI, CT angiographic source images; ASPECTS, Alberta Stroke Program Early CT Score; ICA, internal carotid artery; M1, middle cerebral artery stem (M1 segment); M2, middle cerebral artery post-bifurcation segment (M2 segment); mRS, modified Rankin Scale score.

^a ^Defined as time from stroke onset.

^b ^Denominator is 67 patients who had available follow-up imaging.

The median Mori reperfusion score was 2 (IQR, 1-3). The mean time to reperfusion or procedure termination was 411.2 ± 153.2 minutes. Nineteen patients (27.1%) had a good outcome (mRS ≤ 2) at three months. Twenty-five patients (35.7%) died. The median mRS was 4 (IQR, 2-6).

Independent predictors of good three-month outcome for the entire cohort were Mori reperfusion score (O.R. 11.4, 95% C.I. 2.84-45.5, p < 0.0001), age (O.R. 0.93, 95% C.I. 0.88-0.98, P = 0.0004), and NIHSS score (O.R. 0.79, 95% C.I. 0.64-0.98, p = 0.02). NCCT ASPECTS and CTA-SI ASPECTS were not statistically different between patients with good and poor outcome.

Of sixty-seven patients with post-treatment imaging, hemorrhagic transformation was detected in 39 patients (58.2%): 27 HI1, 8 HI2, 1 PH1 and 3 PH2. There was a 4.5% rate of significant hemorrhagic transformation (PH2), which is within reported rates for IAT[9,11,20]. Of three patients with PH2, one had a left M1 occlusion with mRS of 5, one had a right M1 occlusion with mRS of 3, and one had a right M1 occlusion and died.

### Baseline characteristics and clinical outcome: left- versus right-hemispheric strokes

Table 2 compares left- and right-hemispheric strokes for baseline variables, reperfusion and clinical outcome. Left-sided strokes had higher NIHSS scores (p = 0.02) and lower admission SBP (p = 0.009). The remaining baseline variables were not statistically different, including age, occlusion level, and ASPECTS.

**Table 2:** Left- vs Right-Hemispheric Strokes, Baseline and Post-Treatment Characteristics and Outcome

	Left hemisphere (n = 35)	Right hemisphere (n = 35)	P value	Test
Age, y, mean ± SD	68.9 ± 18.0	65.3 ± 18.9	0.41	Student's T
Female, no. (%)	22 (62.9)	16 (45.7)	0.23	Fisher's exact
Baseline NIHSS score, median (IQR)	20 (17-22)	16 (14-19)	0.02	Mann-Whitney
Admission Glucose, mean ± SD	133.8 ± 35.9	151.1 ± 49.8	0.10	Student's T
Admission SBP, mean ± SD	148.6 ± 30.4	168.4 ± 31.3	0.009	Student's T
Atrial fibrillation, no. (%)	18 (51.4)	14 (40)	0.47	Fisher's exact
Diabetes, no. (%)	8 (22.9)	10 (28.6)	0.79	Fisher's exact
IV tPA treatment, no. (%)	12 (34.3)	17 (48.6)	0.33	Fisher's exact
Time to imaging^a^, minutes, mean ± SD	215.1 ± 142.8	176.2 ± 131.4	0.24	Student's T
Baseline NCCT ASPECTS, median (IQR)	8 (6-8.75)	8 (6-9.75)	0.22	Mann-Whitney
Baseline CTA-SI ASPECTS, median (IQR)	5 (2-7)	5 (3-7)	0.59	Mann-Whitney
ICA:M1:M2, no.	10:20:05	11:20:04	0.92	Chi-square
Time to vessel opening^a^, minutes, mean ± SD	420.0 ± 159.8	402.5 ± 148.1	0.63	Student's T
Mori reperfusion score, median (IQR)	2 (1-3)	2 (1-3)	0.44	Mann-Whitney
Post-treatment hemorrhage, no. (%)	17 (51.5)^b^	22 (62.9)	0.34	Fisher's exact
Good 3-month outcome (mRS ≤ 2), no. (%)	9 (25.7)	10 (28.6)	0.99	Fisher's exact

Abbreviations: NIHSS, National Institutes of Health Stroke Scale; SBP, systolic blood pressure; IV tPA, intravenous tissue plasminogen activator; NCCT, noncontrast head CT; CTA-SI, CT angiographic source images; ASPECTS, Alberta Stroke Program Early CT Score; ICA, internal carotid artery; M1, middle cerebral artery stem (M1 segment); M2, middle cerebral artery post-bifurcation segment (M2 segment); mRS, modified Rankin Scale score.

^a ^Defined as time from stroke onset; ^b ^Denominator is 33 patients who had available follow-up imaging.

There were no significant differences in the degree of reperfusion, clinical outcome and hemorrhage rate between the two hemispheres. The median Mori score for both sides was 2 (IQR, 1-3; P = 0.44). Nine of 35 patients (25.7%) with left-sided strokes versus 10 of 35 patients (28.6%) with right-sided strokes had a good outcome (p = 0.99). Hemorrhage occurred in 17/33 (51.5%) left-sided strokes and 22/35 (62.9%) right-sided strokes (p = 0.46).

### Predictors of outcome: left-versus right-hemispheric strokes

**Figure 1:**
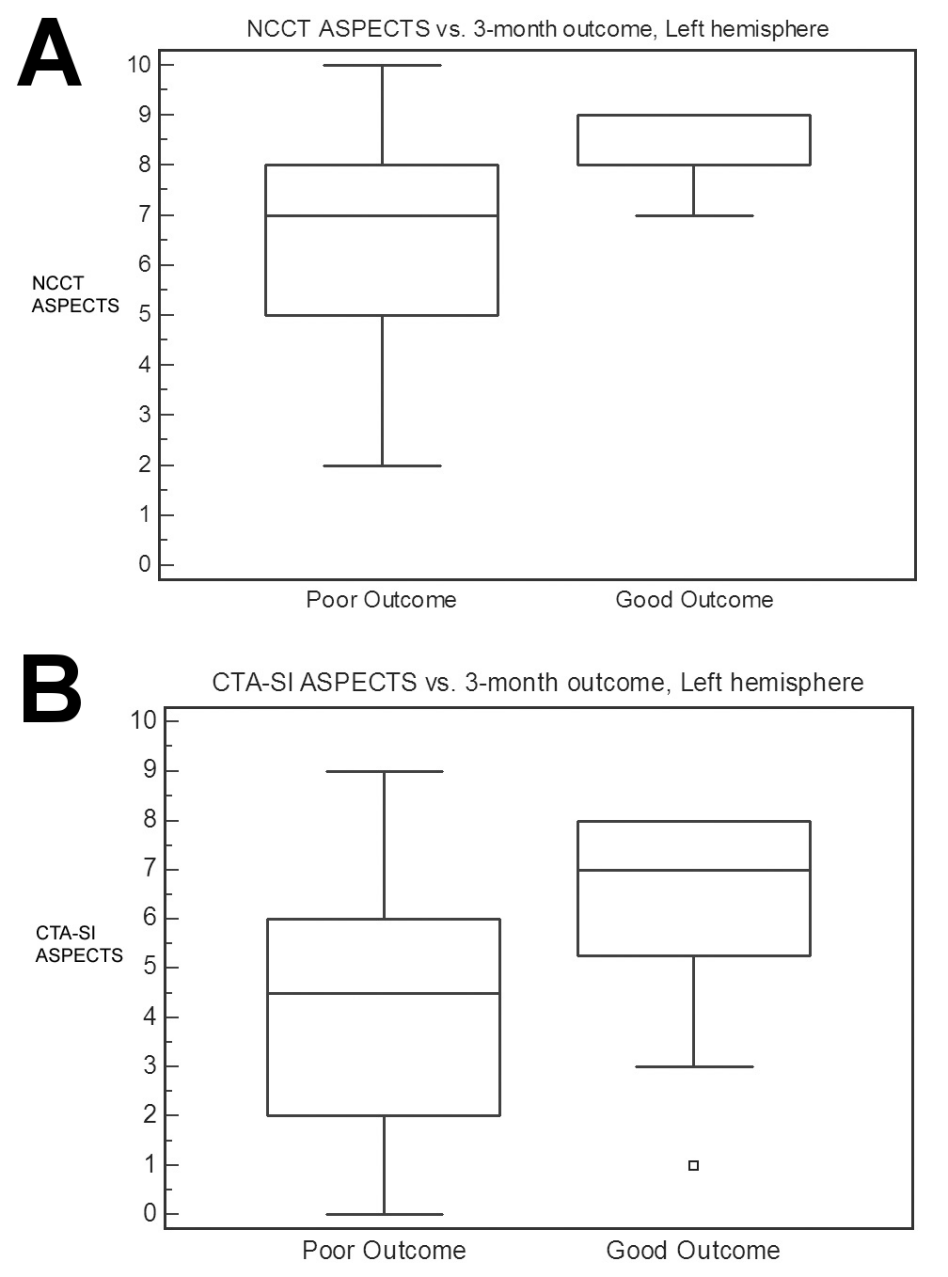
**Left-sided strokes, Box-and-whisker plots**. **A**. Median NCCT ASPECTS for good vs. poor outcome is 8 vs. 7 (P = 0.05). **B**. Median CTA-SI ASPECTS for good vs. poor outcome is 7 vs. 4.5 (P = 0.06). Good outcome is defined as modified Rankin Scale score ≤ 2 at 3 months. Abbreviations: NCCT, noncontrast head CT; CTA-SI, CT angiographic source images; ASPECTS, Alberta Stroke Program Early CT Score.

For left-sided strokes, univariate predictors of outcome were lower NIHSS score (p = 0.0008) and greater reperfusion (p = 0.003, Table 3). There was a strong statistical trend for higher NCCT ASPECTS (p = 0.05) and CTA-SI ASPECTS (p = 0.06) in patients with good outcomes (Figure 1). Independent predictors of good outcome were NIHSS score (O.R. 0.51, 95% C.I. 0.28-0.92, p = 0.0002) and Mori reperfusion score (O.R. 21.3, 95% C.I. 1.13-500, p = 0.006).

**Table 3:** Univariate Predictors of Outcome, Left-Hemispheric Strokes

	Poor, left (n = 26)	Good, left (n = 9)	P value	Test
Age, y, mean ± SD	70.6 ± 17.8	64.1 ± 19.1	0.36	Student's T
Female, no. (%)	14 (53.8)	8 (88.9)	0.11	Fisher's exact
Baseline NIHSS score, median (IQR)	21 (18-22)	14 (9-17)	<0.001	Mann-Whitney
Admission Glucose, mean ± SD	133.8 ± 35.1	133.9 ± 40.3	0.99	Student's T
Admission SBP, mean ± SD	151.7 ± 31.3	139.8 ± 27.3	0.32	Student's T
Atrial fibrillation, no. (%)	13 (50)	5 (55.6)	0.99	Fisher's exact
Diabetes, no. (%)	5 (19.2)	3 (33.3)	0.40	Fisher's exact
IV tPA treatment, no. (%)	8 (30.8)	4 (44.4)	0.69	Fisher's exact
Time to imaging^a^, minutes, mean ± SD	227.2 ± 153.5	180.1 ± 105.4	0.40	Student's T
Baseline NCCT ASPECTS, median (IQR)	7 (5-8)	8 (8-9)	0.05	Mann-Whitney
Baseline CTA-SI ASPECTS, median (IQR)	4.5 (2-6)	7 (5.25-8)	0.06	Mann-Whitney
ICA:M1:M2, no.	8:15:3	2:5:2	0.70	Chi-square
Time to vessel opening^a^, minutes, mean ± SD	435.1 ± 173.8	376.4 ± 105.6	0.35	Student's T
Mori reperfusion score, median (IQR)	2 (0-2)	3 (2-3.25)	0.003	Mann-Whitney
Post-treatment hemorrhage, no. (%)	14 (56)^b^	3 (37.5)^c^	0.44	Fisher's exact

Abbreviations: NIHSS, National Institutes of Health Stroke Scale; SBP, systolic blood pressure; IV tPA, intravenous tissue plasminogen activator; NCCT, noncontrast head CT; CTA-SI, CT angiographic source images; ASPECTS, Alberta Stroke Program Early CT Score; mRS, modified Rankin Scale score.

^a ^Defined as time from stroke onset; ^b ^Denominator is 25 patients who had available follow-up imaging; ^c ^Denominator is 8 patients who had available follow-up imaging.

**Figure 2:**
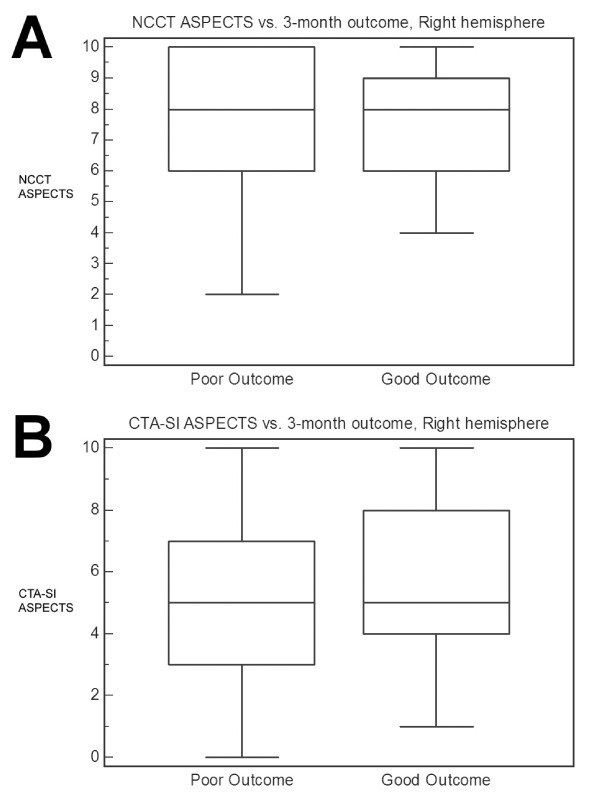
**Right-sided strokes, Box-and-whisker plots**. **A**. Median NCCT ASPECTS for good vs. poor outcome is 8 vs. 8 (P = 0.83). **B**. Median CTA-SI ASPECTS for good vs. poor outcome is 5 vs. 5 (P = 0.66). Good outcome is defined as modified Rankin Scale score ≤ 2 at 3 months. Abbreviations: NCCT, noncontrast head CT; CTA-SI, CT angiographic source images; ASPECTS, Alberta Stroke Program Early CT Score.

For right-sided strokes, univariate predictors of outcome were younger age (p = 0.009) and greater reperfusion (p = 0.006, Table 4). There was no association of pre-treatment ASPECTS with outcome (Figure 2). Independent predictors of good outcome were age (O.R. 0.89, 95% C.I. 0.80-0.98, p = 0.002) and Mori score (O.R. 12.0, 95% C.I. 1.40-100, p = 0.003).

**Table 4:** Univariate Predictors of Outcome, Right-Hemispheric Strokes

	Poor, right (n = 25)	Good, right (n = 10)	P value	Test
Age, y, mean ± SD	70.4 ± 17.8	52.4 ± 15.6	0.009	Student's T
Female, no. (%)	12 (48)	4 (40)	0.72	Fisher's exact
Baseline NIHSS score, median (IQR)	16 (14-20)	15.5 (14-18)	0.73	Mann-Whitney
Admission Glucose, mean ± SD	149.0 ± 54.8	156.3 ± 36.4	0.70	Student's T
Admission SBP, mean ± SD	171.1 ± 33.9	162.1 ± 24.1	0.45	Student's T
Atrial fibrillation, no. (%)	11 (44)	3 (30)	0.70	Fisher's exact
Diabetes, no. (%)	6 (24)	4 (40)	0.42	Fisher's exact
IV tPA treatment, no. (%)	14 (56)	3 (30)	0.26	Fisher's exact
Time to imaging^a^, minutes, mean ± SD	177.8 ± 147.7	172.1 ± 83.6	0.91	Student's T
Baseline NCCT ASPECTS, median (IQR)	8 (6-10)	8 (6-9)	0.83	Mann-Whitney
Baseline CTA-SI ASPECTS, median (IQR)	5 (3-7)	5 (4-8)	0.66	Mann-Whitney
ICA:M1:M2, no.	9:12:4	2:8:0	0.18	Chi-square
Time to vessel opening^a^, minutes, mean ± SD	418.1 ± 158.7	363.4 ± 115.3	0.33	Student's T
Mori reperfusion score, median (IQR)	2 (1-2.25)	3 (3-3)	0.006	Mann-Whitney
Post-treatment hemorrhage, no. (%)	18 (72)	4 (40)	0.12	Fisher's exact

Abbreviations: NIHSS, National Institutes of Health Stroke Scale; SBP, systolic blood pressure; IV tPA, intravenous tissue plasminogen activator; NCCT, noncontrast head CT; CTA-SI, CT angiographic source images; ASPECTS, Alberta Stroke Program Early CT Score; mRS, modified Rankin Scale score.

^a ^Defined as time from stroke onset

## Discussion

In this study of acute ischemic stroke patients with anterior circulation PAO who underwent IAT, the predictors of three-month functional outcome differed by the side of stroke involvement. Namely, for left-sided strokes, baseline NIHSS score, degree of reperfusion, and potentially pre-treatment NCCT ASPECTS predicted outcome, while for right-sided strokes, degree of reperfusion and age were predictors. Despite this finding, there was no significant difference in the rates of good outcome between left- and right-hemispheric strokes.

Importantly, the left- and right-sided stroke cohorts were well matched for major variables previously associated with clinical outcome after IAT, including age[15,17,19], occlusion level[14,27], and recanalization[11,13,14,16,17,19]. Although there was a significant difference in median NIHSS score (another important variable[13,15,19]) of 4 points, this mismatch more likely reflected the inherent bias of the NIHSS for left-hemispheric function rather than a true difference in stroke severity, as the levels of occlusion were nearly identical (p = 0.92), and there were no significant differences in pre-treatment ASPECTS (p = 0.22). Supporting this view is a study which demonstrated that left-sided stroke patients with total anterior circulation syndromes scored 4 points higher on the NIHSS than right-sided stroke patients with larger stroke volumes[4].

Evidence from studies assessing stroke outcome by side of involvement is inconclusive, with some studies demonstrating more favorable outcomes for left-hemispheric strokes and others demonstrating the converse[1,3]. The largest study is the recent retrospective analysis of the VISTA database, which pooled data from stroke trials reporting lateralization[3]. Assessing 1644 placebo-treated patients, the authors found no difference in functional outcome between the two hemispheres, which is in agreement with our findings. The conflicting data may be related to differences in study populations. The previous studies examined patient cohorts encompassing a broad range of stroke severity, including lacunar and total anterior circulation syndromes[1,3]. In comparing left-versus right-hemispheric strokes, most studies did not adjust for occlusion level, which could potentially influence results. Furthermore, the VISTA analysis included posterior circulation strokes as well as 163 intracerebral hemorrhages.

### Clinical Predictors of Outcome

Numerous studies have shown that baseline NIHSS score predicts outcome in stroke patients who receive IV and/or IA therapies[13,15,19,22]. In our study, it was an independent predictor of outcome for the entire cohort. However, when analyzing outcome by side, NIHSS score was an independent predictor of outcome only for left-sided strokes. By contrast, the VISTA analysis demonstrated a significant correlation between NIHSS and outcome for both hemispheres[3]. The likely explanation is that over a broad range of stroke severity (as seen in the VISTA database) NIHSS score is a reasonable surrogate for occlusion level, even for right-sided strokes. For a homogeneous population of large vessel occlusions (as in our study), NIHSS score may not adequately discriminate the amount of territory at risk in the right hemisphere. This last point is supported by numerous imaging studies that have demonstrated that patients with right-sided strokes may have low NIHSS scores despite substantial infarct volumes [4-6].

Similarly, age was an independent predictor of outcome for right-sided but not for left-sided strokes. Age likely emerged as a predictor for the right hemisphere due to the absence of an NIHSS effect. As expected, reperfusion was an independent predictor of outcome for both hemispheres.

If verified in future studies, these findings have significant implications for treatment selection, acute prognostication, and clinical investigation of anterior circulation stroke patients undergoing IAT. Based on our results, using the NIHSS to stratify PAO patients by stroke severity should be done only for left-hemispheric strokes. Right-hemispheric strokes secondary to PAO should be stratified using a clinical scale tailored to the right hemisphere[4].

### Imaging Predictors of Outcome

Neither NCCT nor CTA-SI ASPECTS predicted clinical outcome in the entire patient cohort. However, both ASPECTS scores appeared to predict outcome for left-sided strokes. Patients with left-sided strokes and good outcome had higher NCCT ASPECTS (8 vs. 7, p = 0.05) and CTA-SI ASPECTS (7 vs. 4.5, p = 0.06) scores on pre-treatment imaging than those with poor outcome (Figure 1). No such association between ASPECTS and outcome was seen in right-hemispheric strokes (Figure 2). The lack of a relationship between ischemic burden and outcome in the right hemisphere confirms the findings of a previous study which identified a subset of right-sided stroke patients who had good outcomes despite substantial lesion volumes[6]. No such group was found among the left-sided stroke patients in that study.

Published studies of IAT that have assessed predictors of outcome either have not mentioned the side of involvement[11,14,17-19,28], or have not analyzed left- and right-sided strokes separately [15,16]. Only two studies present their outcomes by the involved hemisphere, although no analysis is performed comparing the two groups[2,16]. This may explain why some studies that have assessed both pre-treatment imaging and outcome have found an association[15,28] and some have not[2,14,16-19]. The difference may depend on the proportion of left-versus right-sided strokes in a particular study.

One plausible explanation for the imaging findings is that language localizes to the left hemisphere in 88-96% of right-handed and 73-4% of left-handed people [29,30]. Furthermore, many anatomic structures responsible for speech are localized to the peri-insular region, including Broca's area[31]. Because of the nature of the pial collateral circulation, the peri-insular region has the most severe reduction in blood flow and progresses to infarction most rapidly with ICA and proximal MCA occlusions. Therefore, in the usually dominant left hemisphere, even modest infarcts will likely have a negative impact on language and clinical outcome, which may explain why smaller infarct burden is associated with better outcome on the left.

While incompletely understood, poor outcomes in right-hemispheric strokes are thought to be related to cognitive deficits, most notably neglect[2,32]. Previous studies of neglect have implicated the right superior temporal gyrus and inferior parietal lobule[33]. Because they lie distally in the MCA territory, an infarct has to be relatively large to involve these regions, and therefore, more infarct burden can be sustained before having a negative clinical effect.

These findings could have significant implications for imaging selection in acute stroke therapy. They highlight the weakness of volumetric-based analysis that does not account for eloquence[34].

### Admission SBP and Hemispheric Lateralization

Our patients with right-hemispheric stroke demonstrated significantly higher mean admission SBP than those with left-hemispheric stroke (168.4 mmHg vs 148.6 mmHg, p = 0.009). This finding was also demonstrated by Meyer et al, who showed that the increase in blood pressure for right-sided strokes was accompanied by significantly increased plasma catecholamine levels[35] and that these changes were mediated by infarction of the insular cortex, the area most at risk in our patients. The autonomic role of the right insular cortex is also supported by a study demonstrating that right insular infarction is associated with elevated serum cardiac troponin T levels[36], mediated by excessive sympathetic activation. It is important to note that there was no significant difference in the admission SBP between our patients with good versus poor outcome.

### Limitations

One limitation of this study is the small number of subjects, particularly those with good outcome. This reflects the highly selected nature of our study population, as well as the generally poor natural history of patients with proximal intracranial artery occlusion. This aspect of the study may have limited our ability to distinguish differences in the rates of good outcome between the two hemispheres. However, despite the small study population, we were able to demonstrate significant differences in predictors of outcome, particularly with respect to the NIHSS. Clearly, our findings need to be verified in larger studies. Additionally, the retrospective nature of this analysis introduces potential biases that should be addressed in prospective studies. While the left- and right-sided strokes in this study had similar stroke severities as reflected by the level of arterial occlusion and the extent of pre-treatment ischemic change on imaging, future studies should incorporate quantitative or semiquantitative assessments of the pial collateral circulation in their analyses.

## Conclusions

In anterior circulation proximal artery strokes treated with IAT, NIHSS predicts outcome only for left-sided strokes, highlighting the need for clinical scales that are more sensitive to right hemispheric function. Additionally, pre-treatment ischemic change on NCCT and CTA source images may be associated with outcome only for the left hemisphere, which has important consequences for using imaging selection to guide therapy. These preliminary findings need to be verified in larger, prospective studies, and may have significant implications for both clinical management and research methodology.

## Abbreviations

AIS: acute ischemic stroke; IAT: intra-arterial reperfusion therapy; ICA: internal carotid artery; MCA: middle cerebral artery; mRS: modified Rankin Scale score; NIHSS: National Institutes of Health Stroke Scale; IQR: interquartile range; SBP: systolic blood pressure; NCCT: noncontrast CT scan; PAO: proximal artery occlusion; CTA: CT angiography; PWI: MRI perfusion-weighted imaging; DWI: diffusion-weighted imaging; IV tPA: intravenous tissue plasminogen activator; ASPECTS: Alberta Stroke Program Early CT Score; CTA-SI: CTA source images; ECASS: European Cooperative Acute Stroke Study

## Competing interests

Albert J. Yoo, James D. Rabinov, Javier Romero, Reza Hakimelahi, R. Gilberto González and Pamela W. Schaefer all have no competing interests. Raul G. Nogueira is a Member of the Physician Advisory Board for Concentric Medical, Inc.; ev3 Neurovascular, Inc.; and Coaxia, Inc. (modest financial compensation). Johnny C. Pryor is a Consultant for ev3 Neurovascular, Inc.; Codman Neurovascular, Inc.; and Micrus, Inc. (no financial compensation). Joshua A. Hirsch is part of the Merci Registry Steering Committee (no financial compensation).

## Authors' contributions

AJY conceived of the study, participated in its design, performed data collection and statistical analysis and drafted the manuscript. JR performed data collection, interpreted the data, and provided critical review of the manuscript. RH performed data collection and statistical analysis and provided critical review of the manuscript.

RGN participated in the study design, data interpretation and critical review of the manuscript. JDR and JCP participated in data collection and critical review of the manuscript. RGG and JAH participated in study design, data interpretation and critical review of the manuscript. PWS conceived of the study, participated in its design, performed data collection and interpretation and provided critical review of the manuscript. All authors read and approved the final manuscript.

## Pre-publication history

The pre-publication history for this paper can be accessed here:

http://www.biomedcentral.com/1471-2377/10/25/prepub
